# Author Correction: ID1 expressing macrophages support cancer cell stemness and limit CD8^+^ T cell infiltration in colorectal cancer

**DOI:** 10.1038/s41467-026-69408-x

**Published:** 2026-02-09

**Authors:** Shuang Shang, Chen Yang, Fei Chen, Ren-shen Xiang, Huan Zhang, Shu-yuan Dai, Jing Liu, Xiao-xi Lv, Cheng Zhang, Xiao-tong Liu, Qi Zhang, Shuai-bing Lu, Jia-wei Song, Jiao-jiao Yu, Ji-chao Zhou, Xiao-wei Zhang, Bing Cui, Ping-ping Li, Sheng-tao Zhu, Hai-zeng Zhang, Fang Hua

**Affiliations:** 1 State Key Laboratory of Bioactive Substance and Function of Natural Medicines, Institute of Materia Medica, Chinese Academy of Medical Sciences & Peking Union Medical College, 100050 Beijing, P. R. China; 2 Beijing Key Laboratory of New Drug Mechanisms and Pharmacological Evaluation Study (BZ0150), Institute of Materia Medica, Chinese Academy of Medical Sciences & Peking Union Medical College, 100050 Beijing, P. R. China; 3 CAMS Key Laboratory of Molecular Mechanism and Target Discovery of Metabolic Disorder and Tumorigenesis, Institute of Materia Medica, Chinese Academy of Medical Sciences & Peking Union Medical College, 100050 Beijing, P. R. China; 4 Department of Colorectal Surgery, National Cancer Center/National Clinical Research Center for Cancer/Cancer Hospital, Chinese Academy of Medical Sciences and Peking Union Medical College, 100021 Beijing, P. R. China; 5 State Key Lab of Molecular Oncology, National Cancer Center/National Clinical Research Center for Cancer/Cancer Hospital, Chinese Academy of Medical Sciences and Peking Union Medical College, 100021 Beijing, P. R. China; 6 Department of Pharmacy, China–Japan Friendship Hospital, 100029 Beijing, P. R. China; 7 Beijing Digestive Diseases Center, Beijing Friendship Hospital, 100050 Beijing, P. R. China; 8 Beijing Key Laboratory for Precancerous Lesion of Digestive Diseases, Beijing Friendship Hospital, 100050 Beijing, P. R. China

**Keywords:** Cancer stem cells, Cancer microenvironment, Tumour immunology, Transcription, Colon cancer

Correction to: *Nature Communications* 10.1038/s41467-023-43548-w, published online 23 November 2023.

In the version of this article initially published, the second representative photo depicting bioluminescence imaging of *Serpinb2*^*KD*^*/Ccl4*^*KD*^—*Id1*^*f/f*^ mice shown in Fig. 5p was incorrect. The image and corresponding statistical analysis in Fig. 5p, and associated source data, are now amended in the HTML and PDF versions of the article. For comparison, the original Fig. 5 is available as Supplementary information accompanying this amendment.

## Supplementary information

Original, uncorrected Fig. 5

