# Supplementary material for: ID1 expressing macrophages support cancer cell stemness and limit CD8+ T cell infiltration in colorectal cancer
Source: Nat Commun. 2023 Nov 23;14:7661. doi: 10.1038/s41467-023-43548-w (PMC10667515; doi:10.1038/s41467-023-43548-w)
Supplement: Supplementary file 1 — Supplementary Information [file 41467_2023_43548_MOESM1_ESM.pdf]

ID1 Expressing Macrophages Support Cancer Cell Stemness and Limit CD8^{+} T Cell Infiltration in Colorectal Cancer

Shuang Shang et. al

Supplementary-figures and legends

Supplementary-tables

Supplementary-figures and legends

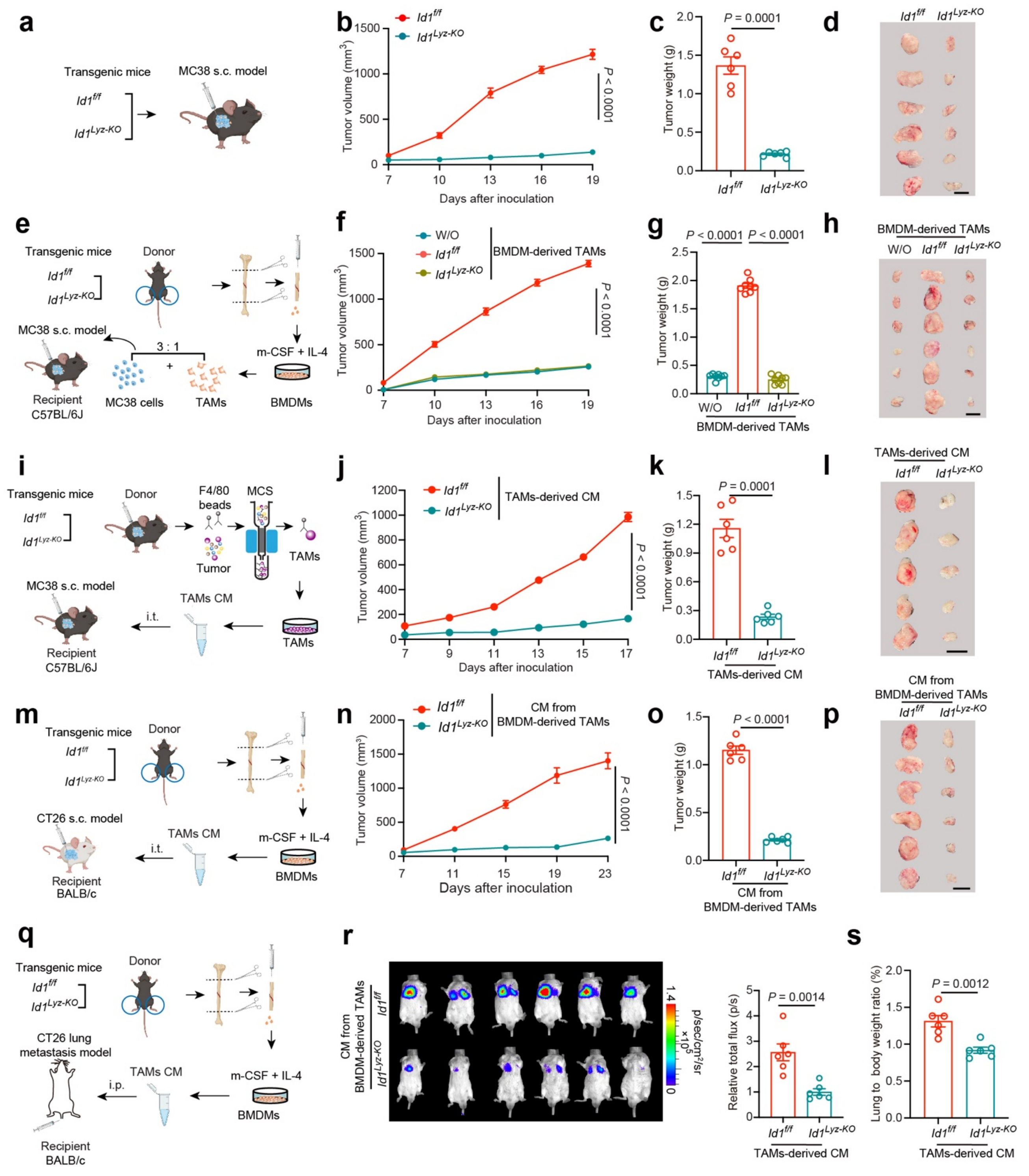

Supplementary Fig. 1 Depletion of *Id1* in TAMs inhibits CRC tumor growth.

a, Schematic diagram for establishing the MC38 s.c. model in *Id1*$^{f/f}$ and *Id1*$^{Lyz\text{-}KO}$ mice. **b-d,** Tumor volumes (**b**), tumor weight (**c**) and representative tumor images (**d**) as presented in (**a**), n = 6 mice per group, Welch's test. Scale bar, 1 cm. **e,** Schematic diagram for adoptive transfer of BMDM-derived TAMs to C57BL/6J mice for establishing

the MC38 and TAMs mixture s.c. model. W/O: without. **f-h,** Tumor volumes (**f**), tumor weight (**g**) and representative tumor images (**h**) of the indicated groups as presented in (**e**), n = 8 mice per group, one-way ANOVA test. Scale bar, 1 cm. **i,** Schematic diagram for establishing the MC38 s.c. model with intratumoral (i.t.) injection of CM from indicated TAMs. **j-l,** Tumor volumes (**j**), tumor weight (**k**) and representative tumor images (**l**) of the indicated groups as presented in (**i**), n = 6 mice per group, Welch's test. Scale bar, 1 cm. **m,** Schematic diagram for establishing the CT26 s.c. model with i.t. injection of CM from BMDM-derived TAMs. **n-p,** Tumor volumes (**n**), tumor weight (**o**) and representative tumor images (**p**) of the indicated groups as presented in (**m**), n = 6 mice per group, Welch's test. Scale bar, 1 cm. **q,** Schematic diagram for establishing the CT26 lung metastasis model in BALB/c mice with i.p. injection of CM from BMDM-derived TAMs. **r,** Representative bioluminescence images and the statistical data of indicated groups as presented in (**q**), n = 6 mice per group, Student's *t* test. **s,** Statistical data of lung weight/body weight ratio of indicated groups as presented in (**q**), n = 6 mice per group, Student's *t* test. Elements of **a, e, i, m** and **q** are created with BioRender.com. Source data are provided as a Source Data file.

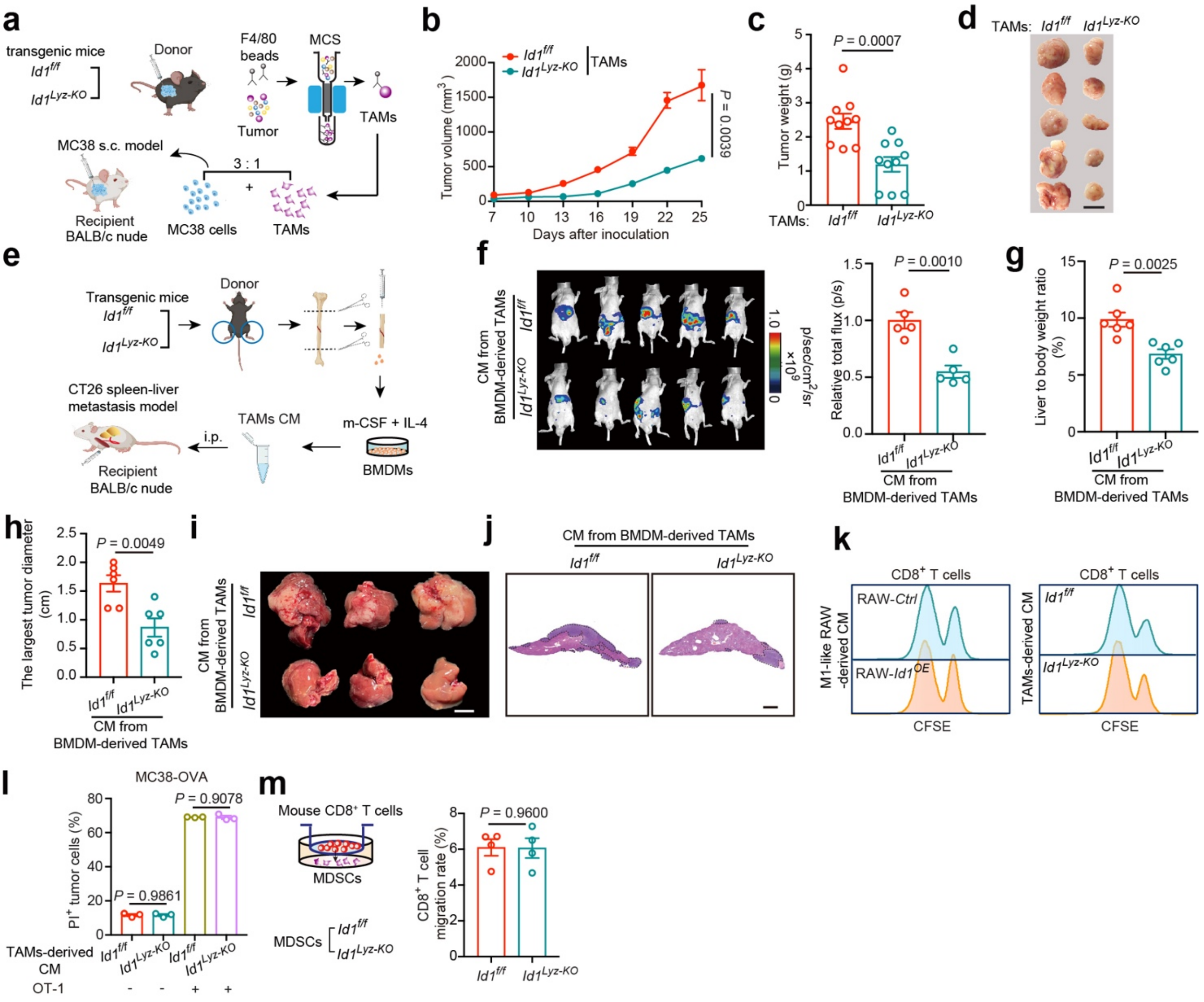

Supplementary Fig. 2 ID1 expressing TAMs promote CRC tumor growth and liver metastasis in immunodeficient mice.

a, Schematic diagram for adoptive transfer of TAMs to BALB/c nude mice for establishing the MC38 s.c. model. **b-d,** Tumor volumes (**b**), tumor weight (**c**) and representative tumor images (**d**) of indicated groups as presented in (**a**), n = 10 mice per group, Student's *t* test. Scale bar, 1 cm. **e,** Schematic diagram for establishing the CT26 spleen-liver metastasis model in BALB/c nude mice with i.p. injection of CM from BMDM-derived TAMs. **f,** Representative bioluminescence images and statistical data of bioluminescence signal of indicated groups as presented in (**e**), n = 5 mice per group, Student's *t* test. **g,** Ratio of liver weight to body weight of indicated groups in (**e**), n = 6 mice per group, Student's *t* test. **h,** The largest diameter of metastatic tumor of the indicated groups presented in (**e**), n = 6 mice per group, Student's *t* test. **i, j,** Representative gross liver images (**i**) and H&E staining of the liver (**j**) for the indicated groups in (**e**). Scale bar, 1 cm (**i**), 2 mm (**j**). **k,** Representative flow cytometry plots of CFSE-labeled $CD8^+$ T cells treated with CM from M1-like *Ctrl* or $Id1^{OE}$ RAW 264.7 cells (left), or with CM

from TAMs isolated from MC38-derived tumor nodules inoculated in *Id1*$^{f/f}$ *or Id1*$^{Lyz\text{-}KO}$ mice, n = 3 biologically independent samples. **l,** Effects of *Id1* depletion in TAMs on OT-1 T cells mediated tumor killing. Results are shown as the percentages of PI$^+$ tumor cells, n = 3 biologically independent samples, Student's *t* test. **m,** Relative migration rate of CD8$^+$ T cells cocultured with *Ctrl* or *Id1*^{KD} MDSCs. n = 4 biologically independent samples, Student's *t* test. Elements of **a** and **e** are created with BioRender.com. Source data are provided as a Source Data file.

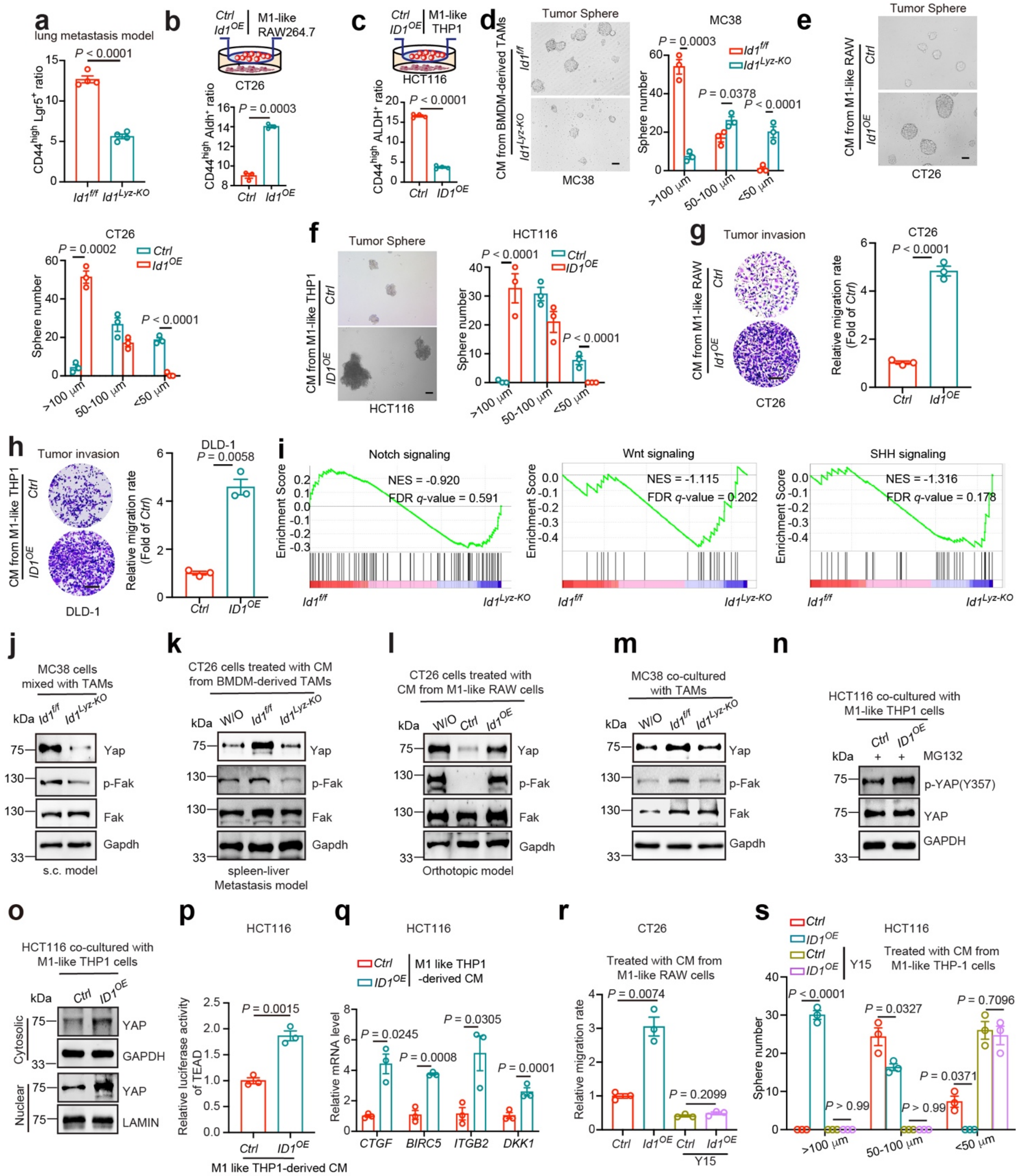

Supplementary Fig. 3 ID1 expressing TAMs promote CRC progression via activating FAK-YAP signaling pathway.

a, CD44^{high} Lgr5^{+} cell ratio in CD45^{-} Epcam^{+} tumor cells from CT26 lung metastasis model presented in Supplementary Fig. 1q. n = 4 biologically independent samples, Student's t test. **b, c,** CD44^{high} Aldh^{+} cell ratio in CT26 cells cocultured with different groups of RAW 264.7 cells (**b**) or in HCT116 cells cocultured with different

groups of THP-1 cells (**c**), n = 3 (**b**) or 4 (**c**) biologically independent samples, Student's *t* test. **d-f,** Images and quantification of MC38 (**d**), CT26 (**e**), or HCT116 (**f**) tumor spheres treated with CM from different groups of BMDMs-derived TAMs (**d**), RAW 264.7 cells (**e**) or THP-1 cells (**f**), n = 3 biologically independent samples, Student's *t* test. **g, h,** Tumor invasiveness of CT26 cells (**g**) or DLD-1 cells (**h**) pre-cultured with CM from different groups of RAW 264.7 cells (**g**) or THP-1 cells (**h**), n = 3 biologically independent samples, Student's *t* test. Scale bar, 100 μm. **i,** GSEA analysis on differentially expressed genes between $CD45^-$ $Epcam^+$ tumor cells of groups presented in Fig.4g with a predefined gene set of Notch, Wnt and SHH signaling. n = 3 biologically independent samples per group in the RNA-seq data. **j-l,** Immunoblots of indicated proteins in $CD45^-$ $Epcam^+$ tumor cells isolated from tumors nodules as presented in Fig. 2a (**j**), 2e (**k**) and 2p (**l**), n = 3 biologically independent samples. **m,** Immunoblots of indicated proteins in MC38 cells cocultured with TAMs isolated from MC38 derived tumors inoculated in $Id1^{f/f}$ or $Id1^{Lyz\text{-}KO}$ mice, n = 3 biologically independent samples. **n,** Immunoblots of indicated proteins in HCT116 cells cocultured with different groups of THP-1 cells in addition of MG132, n = 3 biologically independent samples. **o,** Immunoblots of cytosolic and nuclear YAP in HCT116 cells cocultured with different groups of THP-1 cells, n = 3 biologically independent samples. **p,** Relative luciferase activity of TEAD in HCT116 cells cocultured with different groups of THP-1 cells, *n* = 3 biologically independent samples, Student's *t* test. **q,** Relative mRNA expression of YAP downstream genes in HCT116 cells treated with different groups of THP-1 cells, *n* = 3 biologically independent samples, Student's *t* test. **r,** Effects of Y15 on the invasiveness of CT26 cells pre-treated with CM from M1-like *Ctrl* or $Id1^{OE}$ RAW 264.7 cells, n = 3, Brown-Forsythe ANOVA test. Scale bar, 100 μm. **s,** Effects of Y15 on tumor sphere formation ability of HCT116 cells treated with CM from M1-like *Ctrl* or $ID1^{OE}$ THP-1 cells, n = 3, Student's *t* test. Source data are provided as a Source Data file.

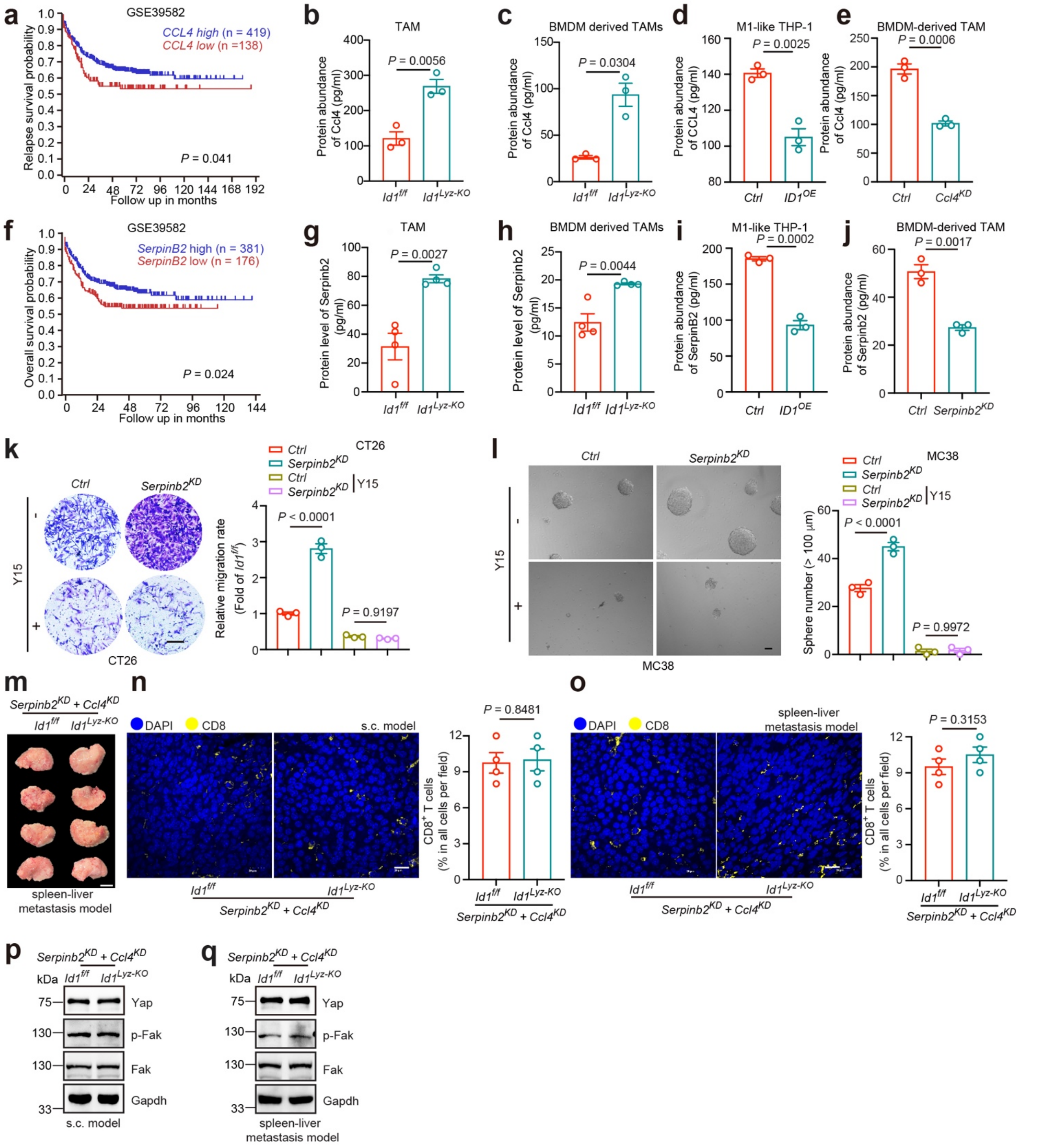

Supplementary Fig. 4 ID1 inhibits *CCL4* and *SerpinB2* transcription in TAMs to promote CRC development. **a,** Kaplan-Meier survival plot stratified by *CCL4* expression of CRC patients (https://hgserver1.amc.nl/cgi-bin/r2/main.cgi). **b,** Ccl4 abundance in the CM from *Id1*$^{f/f}$ and *Id1*$^{Lyz\text{-}KO}$ TAMs, n = 3 biologically independent samples, Welch's test. **c,** Ccl4 abundance in the CM from BMDM-derived *Id1*$^{f/f}$ or *Id1*$^{Lyz\text{-}KO}$ TAMs, n = 3 biologically independent samples, Welch's test. **d,** CCL4 abundance in M1-like *Ctrl* or *ID1*^{OE} THP-1 cells, n = 3 biologically independent samples, Student's *t* test. **e,** Ccl4 abundance in the CM from BMDM-derived TAMs infected with

lentivirus expressing *Ccl4*-specific shRNA, n = 3 biologically independent samples, Student's *t* test. **f,** Kaplan-Meier survival plot stratified by *SerpinB2* expression of CRC patients (https://hgserver1.amc.nl/cgi-bin/r2/main.cgi). **g,** Serpinb2 abundance in the CM from $Id1^{f/f}$ and $Id1^{Lyz\text{-}KO}$ TAMs, n = 4 biologically independent samples, Student's *t* test. **h,** Serpinb2 abundance in BMDM-derived $Id1^{f/f}$ or $Id1^{Lyz\text{-}KO}$ TAMs, n = 4 biologically independent samples, Welch's test. **i,** SerpinB2 abundance in M1-like *Ctrl* or $ID1^{OE}$ THP-1 cells, n = 3 biologically independent samples, Student's *t* test. **j,** Serpinb2 abundance in the CM from BMDM-derived TAMs infected with lentivirus expressing *Serpinb2*-specific shRNA, n = 3 biologically independent samples, Student's *t* test. **k,** Y15 on CT26 invasiveness pretreated with CM from *Ctrl* or $Serpinb2^{KD}$ BMDM-derived TAMs. n = 3 biologically independent samples, one-way ANOVA test. Scale bar, 100 μm. **l,** Y15 on tumor sphere forming ability of MC38 cells treated with CM from *Ctrl* or $Serpinb2^{KD}$ BMDM-derived TAMs. n = 3 biologically independent samples, one-way ANOVA test. Scale bar, 100 μm. **m,** Representative gross images of liver in the indicated groups as presented in Fig. 5p. Scale bar, 1 cm. **n, o,** Representative mIHC images and statistical data of $CD8^+$ T cells infiltrated in the tumor tissues of s.c. model as presented in Fig. 5n (**n**) and spleen-liver metastasis model (**o**) as presented in Fig. 5p, n = 4 biologically independent samples, Student's *t* test. **p, q,** Immunoblots of Yap, p-Fak and Fak in $CD45^-$ $EpCAM^+$ tumor cells isolated from the indicated groups of tumor tissues in the s.c. model as presented in Fig. 5n (**p**) or in the spleen-liver metastasis model as presented in Fig. 5p (**q**), n = 3 biologically independent samples. Source data are provided as a Source Data file.

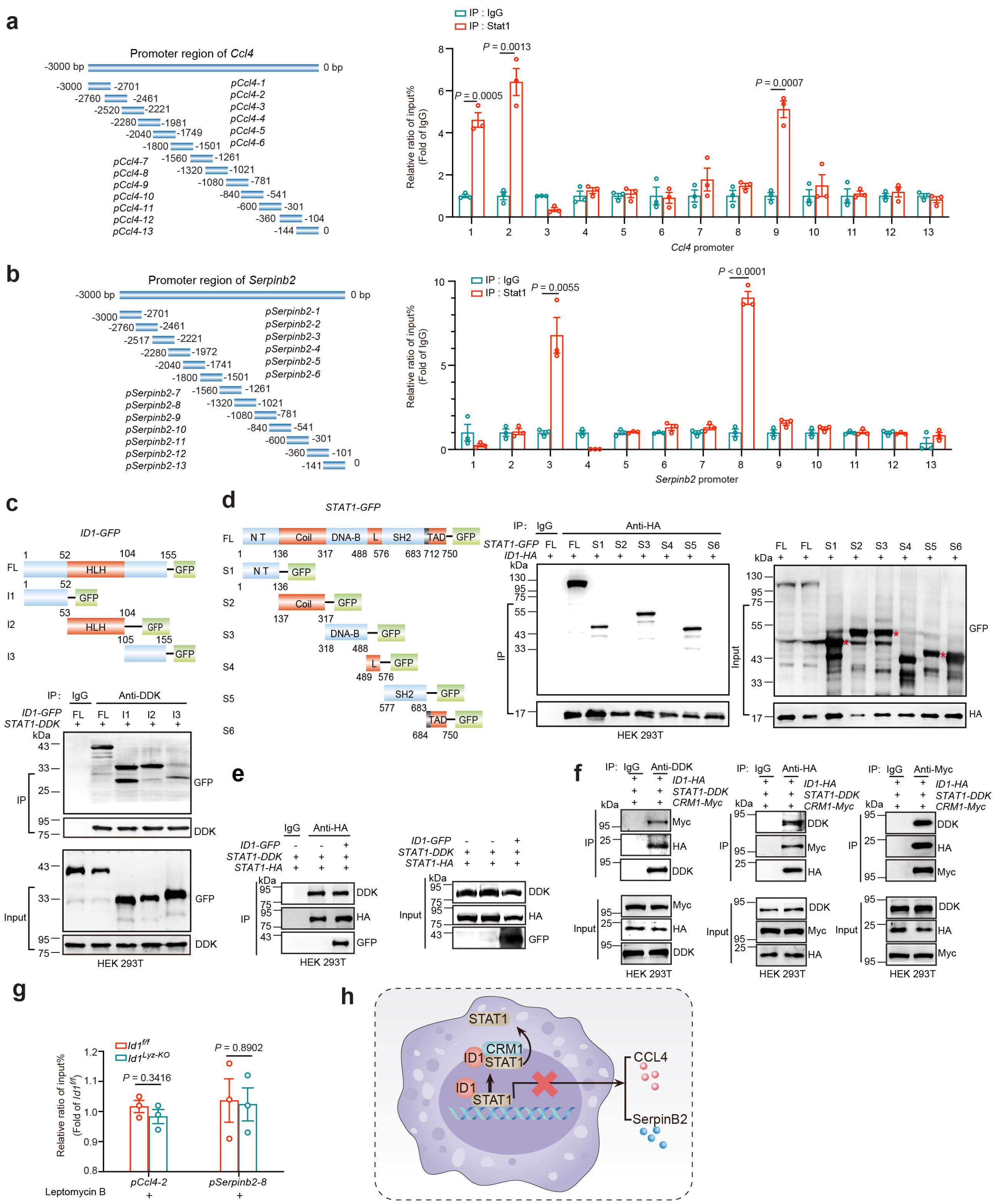

Supplementary Fig. 5 ID1 interacts with STAT1 to promote the formation of ID1-STAT1-CRM1 heterotrimeric complex and inhibit STAT1-mediated *CCL4* and *SerpinB2* transcription.

a, b, Identification of the Stat1 binding region on *Ccl4* (**a**) and *Serpinb2* (**b**) promoter. RAW264.7 cells were subjected to chromatin immunoprecipitation (ChIP) using antibodies against Stat1. IgG isotype antibody was used

as control, n = 3 biologically independent samples, Student's *t* test. **c,** Mapping ID1 regions involved in STAT1 binding. Up: Schematic diagram of GFP-tagged *ID1* deletion mutants. Down: Cell extracts from HEK 293T cells transfected with indicated constructs were immunoprecipitated with anti-DDK antibody and blotted with anti-GFP antibody. IgG isotype antibody was used as control. **d,** Mapping STAT1 regions involved in ID1 binding. Left: Schematic diagram of GFP-tagged *STAT1* deletion mutants. Right: Cell extracts from HEK 293T cells transfected with indicated constructs were immunoprecipitated with anti-HA antibody and blotted with anti-GFP antibody. IgG isotype antibody was used as control, n = 3 biologically independent samples. **e,** Co-immunoprecipitation to detect the effect of ID1 on the homodimerization of STAT1, n = 3 biologically independent samples. **f,** Cell extracts from HEK 293T cells co-transfected with indicated expressing plasmids were immunoprecipitated with anti-DDK, anti-HA or anti-Myc antibodies respectively. IgG isotype antibody was used as control, n = 3 biologically independent samples. **g,** $Id1^{f/f}$ and $Id1^{Lyz\text{-}KO}$ TAMs under the treatment of leptomycin B (100 nM) for 6 hours were subjected to ChIP using antibodies against Stat1. Data are presented as the proportion of chromatin input (input%), n = 3 biologically independent samples, Student's *t* test. **h,** Cartoon showing the proposed molecular mechanism of ID1 in the inhibition of *CCL4* and *SerpinB2* transcription. ID1 recruits CRM1 to STAT1 to form a heterotrimeric protein complex, which promotes STAT1 cytoplasmic distribution and inhibits the transcription of *CCL4* and *SerpinB2*. *Elements adapted from Tsilimigras DI, et al. Liver metastases. Nat Rev Dis Primers. 7:27, 2021; are reproduced with permission from Springer Nature (https://www.nature.com/nrmp/)*[56]. Source data are provided as a Source Data file.

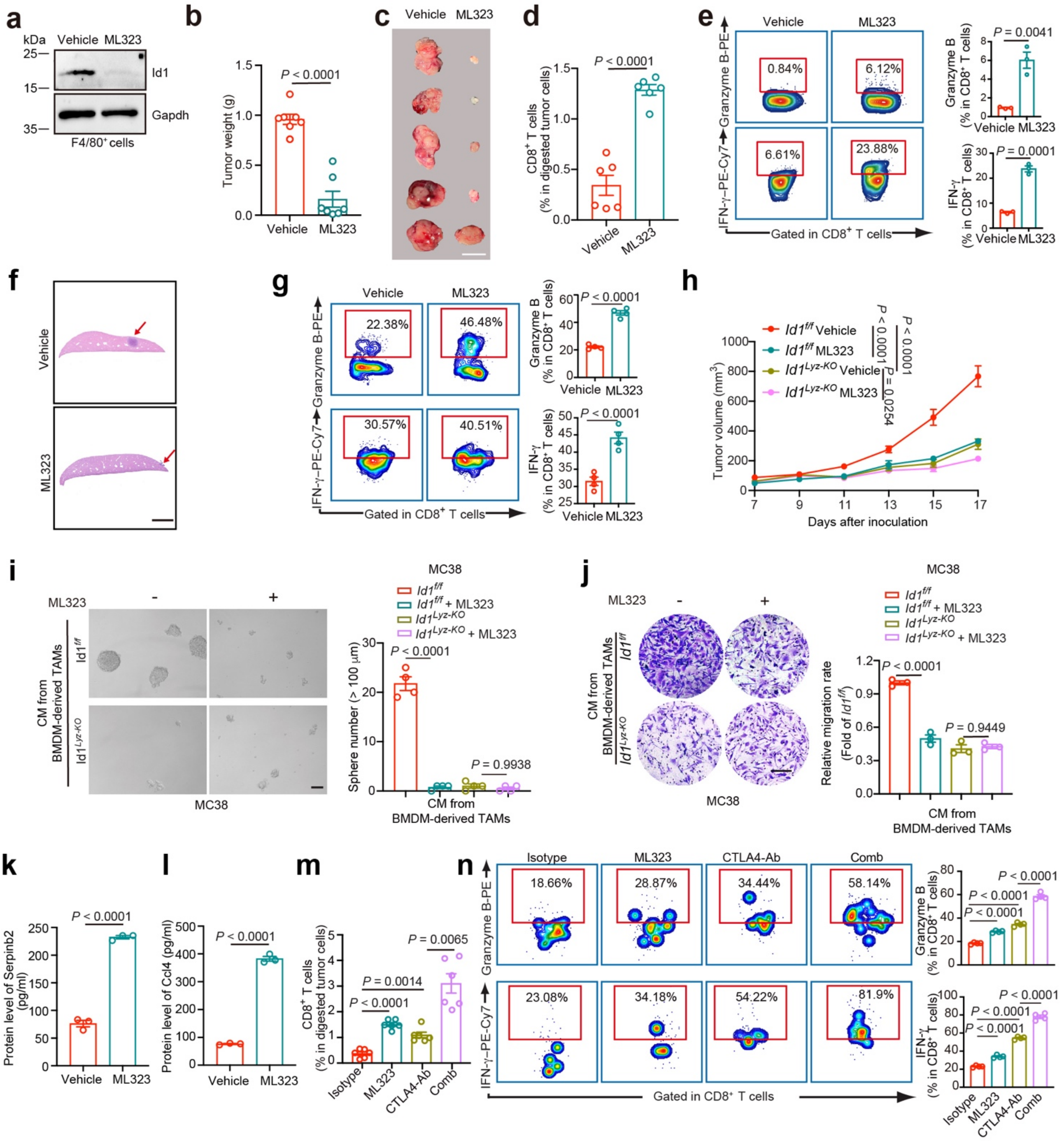

Supplementary Fig. 6 Targeting ID1 in TAMs Inhibits CRC Progression and Sensitizes Tumor Cells to Immunotherapy.

a, Immunoblot of Id1 in F4/80^{+} TAMs in the indicated groups as presented in Fig. 7a, n = 3 biologically independent samples. **b, c,** Tumor weight (**b**) and representative tumor nodule images (**c**) of indicated groups as presented in Fig. 7a, *n* = 8 mice per group, Welch's test. Scale bar, 1 cm. **d,** The percentage of CD8^{+} T cells infiltrated in the indicated tumor tissues as presented in Fig. 7a, *n* = 6 mice per group, Student's *t* test. **e,** Representative density dot plots and the statistical data for IFN-γ^{+} CD8^{+} T cells and Granzyme B^{+} CD8^{+} T cells in the tumor tissues from the indicated

groups as presented in Fig. 7a, *n* = 3 mice per group, Student's *t* test. **f,** Representative H&E staining of the liver from the indicated groups as presented in Fig. 7e. Scale bar, 2 mm. **g,** Representative density dot plots and the statistical data for IFN-γ^+ $CD8^+$ T cells and Granzyme B^+ $CD8^+$ T cells in liver metastases from the indicated groups as presented in Fig. 7e, *n* = 4 mice per group, Student's *t* test. **h,** Tumor growth of indicated groups presented in Fig. 7j. **i,** ML323 treatment on tumor sphere forming ability of MC38 cells treated with CM from $Id1^{f/f}$ *or* $Id1^{Lyz\text{-}KO}$ BMDM-derived TAMs, n = 4 biologically independent samples, one-way ANOVA test. Scale bar, 100 μm. **j,** ML323 treatment on tumor invasiveness of MC38 cells pre-treated with CM from $Id1^{f/f}$ or $Id1^{Lyz\text{-}KO}$ BMDM-derived TAMs, n = 3 biologically independent samples, one-way ANOVA test. Scale bar, 100 μm. **k, l,** Protein level of Serpinb2 and Ccl4 in the CM of TAMs isolated as presebted in Fig. 7l, *n* = 3 biologically independent samples, Student's *t* test. **m,** The percentages of infiltrating $CD8^+$ T cells in the indicated tumor tissues as presented in Fig. 7w, *n* = 6 mice per group, Welch's test. **n,** Representative density dot plots and the statistical data for IFN-γ^+ $CD8^+$ T cells and Granzyme B^+ $CD8^+$ T cells in the tumor tissues from the indicated groups as presented in Fig. 7w, *n* = 4 mice per group, Welch's test. Source data are provided as a Source Data file.

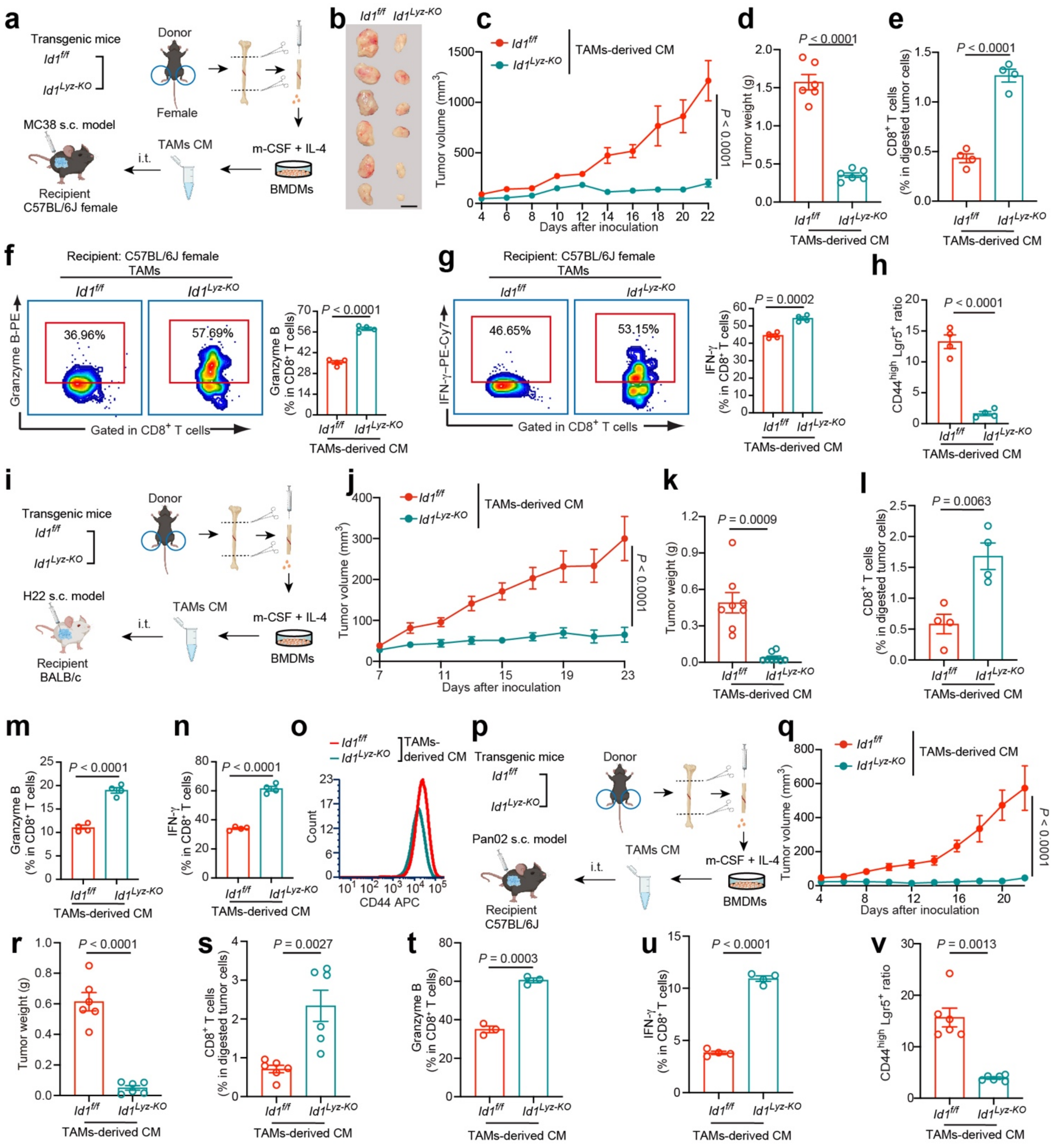

Supplementary Fig. 7 The generalizability of *Id1* depletion in TAMs in the inhibition of tumor growth. **a,** Schematic diagram for establishing the female MC38 s.c. model with i.t. injection of CM from female $Id1^{f/f}$ or $Id1^{Lyz\text{-}KO}$ BMDM-derived TAMs. **b-d,** Representative tumor images (**b**), tumor volumes (**c**) and tumor weight (**d**) of the indicated groups as presented in (**a**), n = 6 mice per group, Student's *t* test. Scale bar, 1 cm. **e,** Percentage of $CD8^+$ T cells infiltrated in the tumor tissues as presented in (**a**), n = 4 mice per group, Student's *t* test. **f, g,** Representative density dot plots and the statistical data for Granzyme B^+ $CD8^+$ T cells and IFN-γ^+ $CD8^+$ T cells in the tumor tissues from the indicated groups as presented in (**a**), *n* = 4 biologically independent samples per group,

Student's *t* test. **h,** $CD44^{high}$ $Lgr5^{+}$ cell ratio in $CD45^{-}$ $Epcam^{+}$ tumor cells from the indicated groups as presented in (**a**), $n = 4$ biologically independent samples, Student's *t* test. **i,** Schematic diagram for establishing the H22 s.c. model with i.t. injection of CM from $Id1^{f/f}$ or $Id1^{Lyz\text{-}KO}$ BMDM-derived TAMs. **j, k,** Tumor volumes (**j**) and tumor weight (**k**) of the indicated groups as presented in (**i**), n = 8 mice per group, Student's *t* test. Scale bar, 1 cm. **l,** Percentage of $CD8^{+}$ T cells infiltrated in the tumor tissues from recipient mice in (**i**), n = 4 mice per group, Student's *t* test. **m, n,** Statistical data for Granzyme B^{+} $CD8^{+}$ T cells and IFN-γ^{+} $CD8^{+}$ T cells in the tumor tissues from the indicated groups as presented in (**i**), $n = 4$ biologically independent samples per group, Student's *t* test. **o,** CD44 in $CD45^{-}$ $Epcam^{+}$ tumor cells in the indicated groups as presented in (**i**). **p,** Schematic diagram for establishing the Pan02 s.c. model with i.t. injection of CM from $Id1^{f/f}$ or $Id1^{Lyz\text{-}KO}$ BMDM-derived TAMs. **q, r,** Tumor volumes (**q**) and tumor weight (**r**) of the indicated groups as presented in (**p**), n = 6 mice per group, Student's *t* test. Scale bar, 1 cm. **s,** Percentage of $CD8^{+}$ T cells infiltrated in the tumor tissues as presented in (**p**), n = 6 mice per group, Student's *t* test. **t, u,** The percentage of Granzyme B^{+} $CD8^{+}$ T cells and IFN-γ^{+} $CD8^{+}$ T cells in the tumor tissues from the indicated groups as presented in (**p**), $n = 3$ (**t**) or 4 (**u**) mice per group, Student's *t* test. **v,** $CD44^{high}$ $Lgr5^{+}$ cell ratio in $CD45^{-}$ $Epcam^{+}$ tumor cells from the indicated groups as presented in (**p**). Elements of **a, i** and **p** are created with BioRender.com. Source data are provided as a Source Data file.

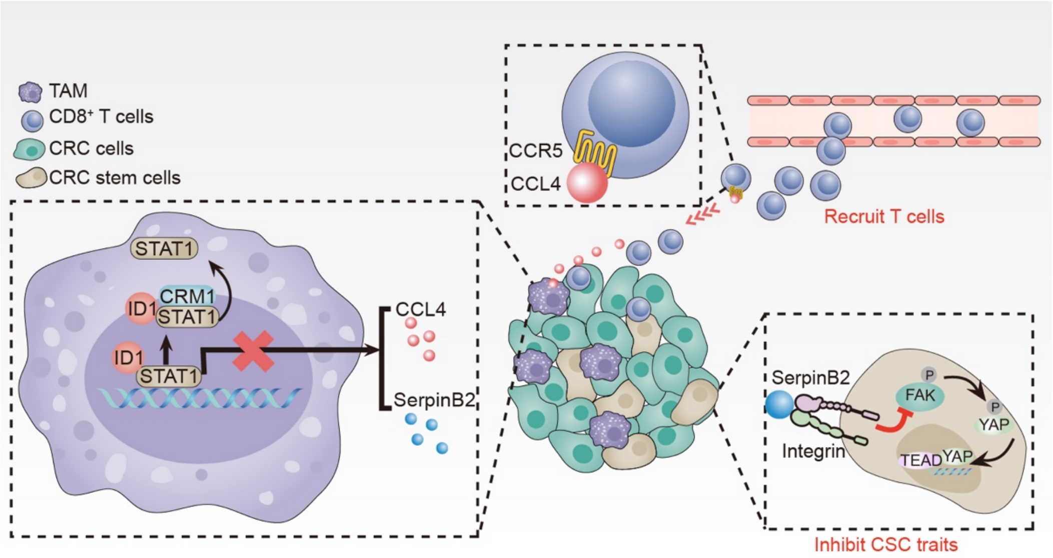

Supplementary Fig. 8 Graphical model.

ID1 interacts with STAT1 to promote STAT1 cytoplasmic distribution via enhancing its association with CRM1, which reduces STAT1-mediated transcription of *CCL4* and *SerpinB2* in TAMs. The reduced CCL4 limits $CD8^+$ T cells recruitment to tumor sites, while the diminished SerpinB2 enhances CRC stemness traits via expanding FAK-YAP signaling activity. Such two effects synergistically promote CRC growth and metastasis. *Elements adapted from Tsilimigras DI, et al. Liver metastases. Nat Rev Dis Primers. 7:27, 2021; are reproduced with permission from Springer Nature (https://www.nature.com/nrmp/)*[56].

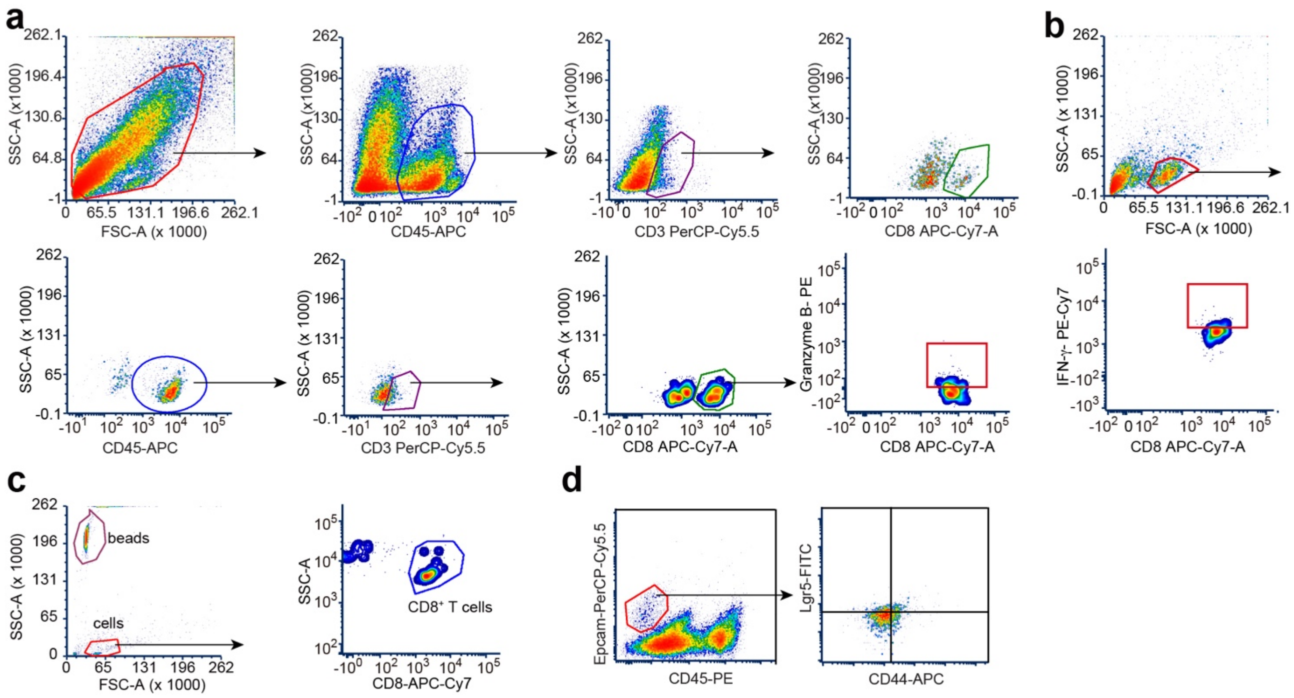

Supplementary Fig. 9 Flow cytometry gating strategy for all stains.

a, Gating strategy of CD8$^+$ T cells in tumor tissues (Fig. 3b, 3g and 7h; Supplementary Fig. 6d, 6m, 7e, 7l and 7s). Among all cells, leukocytes were gated as CD45-APC$^+$, lymphocytes were gated as CD3-PerCP-Cy5.5$^+$, effective T cells were gated as CD8-APC-Cy7$^+$. **b,** Gating strategy of CD8$^+$/Granzyme B or CD8$^+$/IFN-γ T cells in tumor cells suspension after ficoll separation. The first gate excludes nonlymphocyte populations based on forward and side scatter (FSC and SSC). Then leukocytes were gated as CD45-APC$^+$, lymphocytes were gated as CD3-PerCP-Cy5.5$^+$, effective T cells were gated as CD8-APC-Cy7$^+$, active CD8$^+$ T cells were gated by Granzyme B-PE and IFN-γ-PE-Cy7 (Fig. 3d; Supplementary Fig. 6e, 6g, 6n, 7f, 7g, 7m, 7n, 7t and 7u). **c,** Gating strategy of CD8$^+$ T cells migration assay. effective T cells were gated as CD8-APC-Cy7$^+$ (Fig. 3o, 3p, 3q, 5g, 7m and 7o; Supplementary Fig. 2m). **d,** Gating strategy of tumor cells (Fig. 4a, 4b, 7d and 7i; Supplementary Fig. 3a, 7h, 7o and 7v). Among all cells in tumor tissues, tumor cells were gated by Epcam-Percp-Cy5.5$^+$/CD45$^-$.

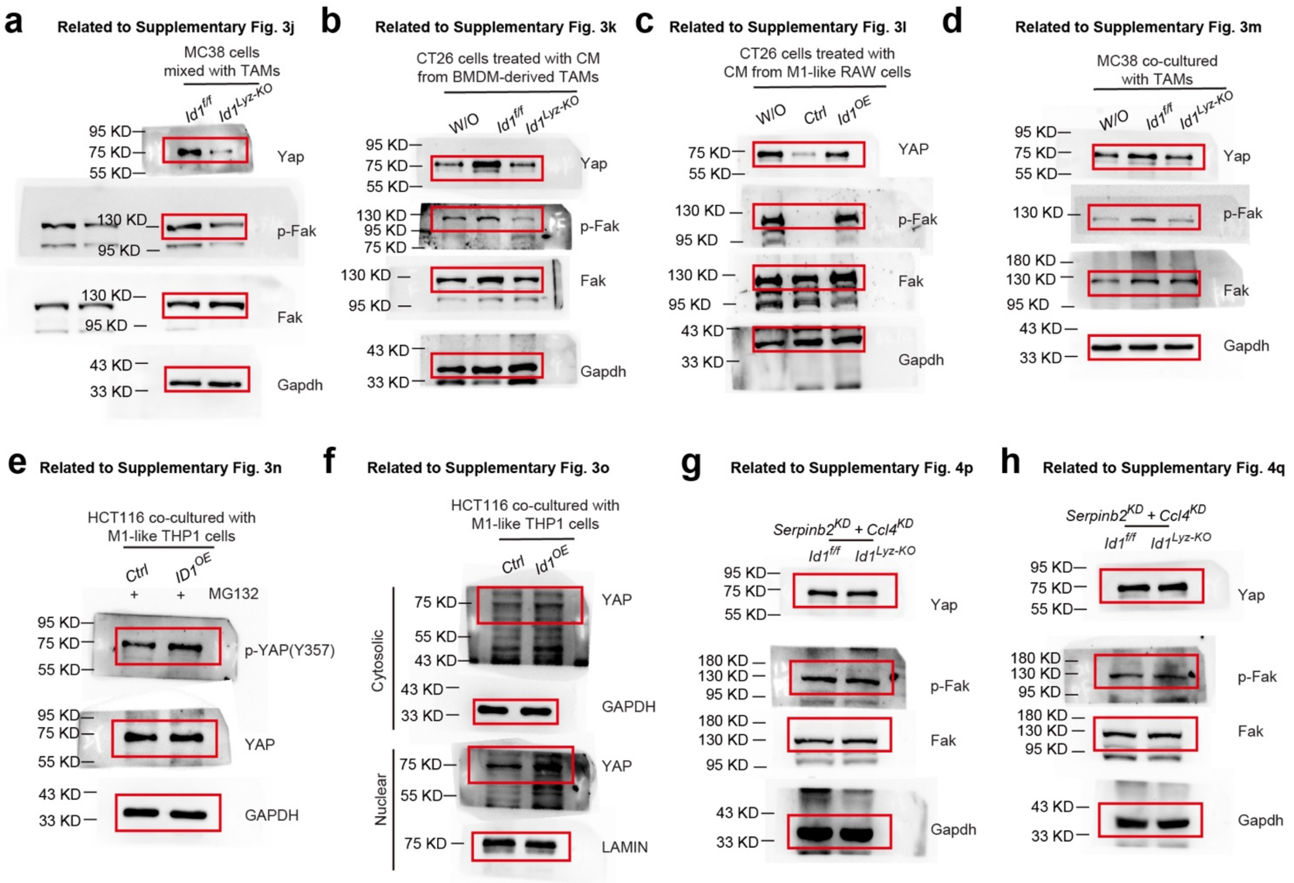

Supplementary Fig. 10 Uncropped images of Western blot in Supplementary Fig. 3, 4.

a-h, Uncropped images of Western blot in Supplementary Fig. 3j, 3k, 3l, 3m, 3n, 3o, 4p and 4q.

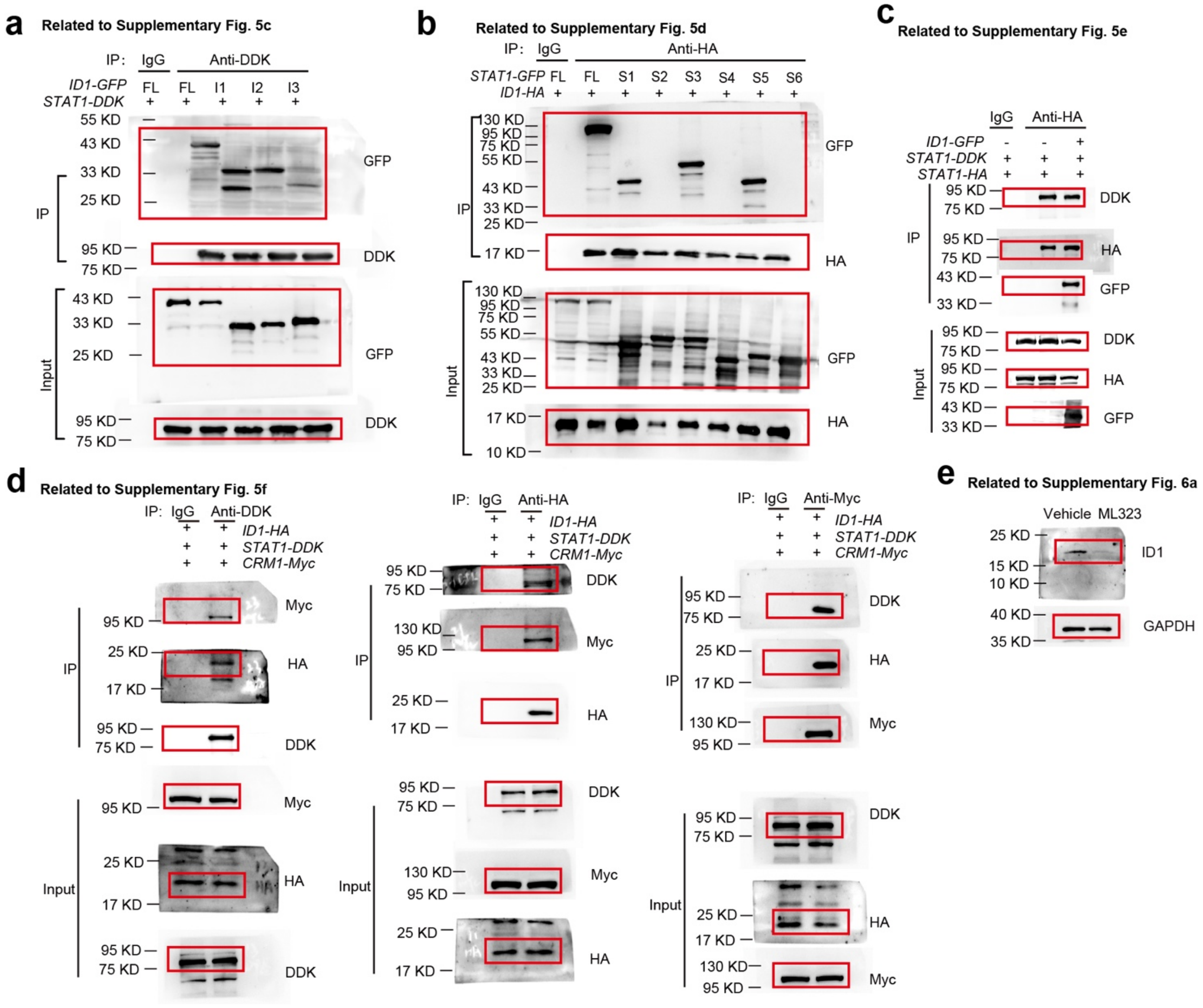

Supplementary Fig. 11 Uncropped images of Western blot in Supplementary Fig. 5, 6.

a-e, Uncropped images of Western blot in Supplementary Fig. 5c, 5d, 5e, 5f and 6a.

Supplementary-tables

Supplementary Table 1 The relationship between ID1 expressing TAMs and clinicopathological features of CRC patients.

Clinicopathological features	Cases	ID1^{+} TAM counts		$\chi 2$	P
		Low	High		
Age (years)					
< 68	49	25	24	0.087	0.6750
≥ 68	52	25	27		
Sex					
female	51	24	27	0.4866	0.4854
male	50	27	23		
Lymph node metastasis					
0	61	36	25	4.474	0.0344
≥ 1	40	15	25		
Pathological grade					
I-II	84	45	39	5.109	0.0238
III-IV	17	4	13		
TNM stage					
1-2	61	37	24	5.186	0.0228
3-4	40	15	25		

Chi-square test was used in the statistics.

Supplementary Table 2 Clinical information of the CRC patients.

Patients Number	Sample specimen	Age	Sex
1	Tumor	50~60	M
2	Tumor	50~60	M
3	Tumor	50~60	M
4	Tumor	60~70	F
5	Tumor	60~70	F
6	PBMC	40~50	M
7	PBMC	60~70	F
8	PBMC	40~50	F
9	PBMC	50~60	M
