# Supplementary material for: ID1 expressing macrophages support cancer cell stemness and limit CD8+ T cell infiltration in colorectal cancer
Source: Nat Commun. 2023 Nov 23;14:7661. doi: 10.1038/s41467-023-43548-w (PMC10667515; doi:10.1038/s41467-023-43548-w)
Supplement: Supplementary file 3 — Reporting Summary [file 41467_2023_43548_MOESM3_ESM.pdf]

nature portfolio

Corresponding author(s): Fang Hua

Last updated by author(s): Oct 31, 2023

Reporting Summary

Nature Portfolio wishes to improve the reproducibility of the work that we publish. This form provides structure for consistency and transparency in reporting. For further information on Nature Portfolio policies, see our Editorial Policies and the Editorial Policy Checklist.

Statistics

For all statistical analyses, confirm that the following items are present in the figure legend, table legend, main text, or Methods section.

n/a	Confirmed	
☐	☒	The exact sample size (*n*) for each experimental group/condition, given as a discrete number and unit of measurement
☐	☒	A statement on whether measurements were taken from distinct samples or whether the same sample was measured repeatedly
☐	☒	The statistical test(s) used AND whether they are one- or two-sided<br>*Only common tests should be described solely by name; describe more complex techniques in the Methods section.*
☐	☒	A description of all covariates tested
☐	☒	A description of any assumptions or corrections, such as tests of normality and adjustment for multiple comparisons
☐	☒	A full description of the statistical parameters including central tendency (e.g. means) or other basic estimates (e.g. regression coefficient) AND variation (e.g. standard deviation) or associated estimates of uncertainty (e.g. confidence intervals)
☐	☒	For null hypothesis testing, the test statistic (e.g. *F*, *t*, *r*) with confidence intervals, effect sizes, degrees of freedom and *P* value noted<br>*Give P values as exact values whenever suitable.*
☒	☐	For Bayesian analysis, information on the choice of priors and Markov chain Monte Carlo settings
☒	☐	For hierarchical and complex designs, identification of the appropriate level for tests and full reporting of outcomes
☒	☐	Estimates of effect sizes (e.g. Cohen's *d*, Pearson's *r*), indicating how they were calculated

Our web collection on statistics for biologists contains articles on many of the points above.

Software and code

Policy information about availability of computer code

Data collection	FACSuite, OLYMPUS FC31S, LineGene 9620, BioTek Synergy H1
Data analysis	GraphPad Prism 8, Image-Pro Plus 6.0, FCS Express 6 Flow Research Edition, Gel Pro Analyzer 6.0

For manuscripts utilizing custom algorithms or software that are central to the research but not yet described in published literature, software must be made available to editors and reviewers. We strongly encourage code deposition in a community repository (e.g. GitHub). See the Nature Portfolio guidelines for submitting code & software for further information.

Data

Policy information about availability of data

All manuscripts must include a data availability statement. This statement should provide the following information, where applicable:

- Accession codes, unique identifiers, or web links for publicly available datasets
- A description of any restrictions on data availability
- For clinical datasets or third party data, please ensure that the statement adheres to our policy

The data that support the findings of this study are available from the corresponding authors. The data generated in this study are available in the paper, supplementary information. Source data are provided with this paper.
Raw and processed RNA-seq data have been deposited to GEO, including GSE188572 (https://www.ncbi.nlm.nih.gov/geo/query/acc.cgi?acc=GSE188572) and GSE200855 (https://www.ncbi.nlm.nih.gov/geo/query/acc.cgi?acc=GSE200855). The proteome data have been deposited to iProX, https://www.iprox.cn//page/

project.html?id=IPX0007320000.

Research involving human participants, their data, or biological material

Policy information about studies with human participants or human data. See also policy information about sex, gender (identity/presentation), and sexual orientation and race, ethnicity and racism.

Reporting on sex and gender	Donors were 50% female and 50% male in the CRC microarray data, 44% female and 56% male in the fresh tumor or PBMC samples. Gender information was not reported.
Reporting on race, ethnicity, or other socially relevant groupings	The human CRC tumor tissues in the microarray was purchased from Shanghai Outdo Biotech. The human CRC tumor or PBMC samples were obtained from the Cancer Hospital, Chinese Academy of Medical Sciences and Peking Union Medical College. All the individuals are primarily Asian ancestry.
Population characteristics	The patients were all diagnosed as colon cancer.
Recruitment	There is no specific inclusion criteria in the commercial CRC tissue microarray. In the recruitment of CRC patients from Cancer Hospital, Chinese Academy of Medical Sciences and Peking Union Medical College, patients without chronic diseases among the age of 40~70 were enrolled.
Ethics oversight	The CRC tumor samples were collected with the approved consent by the institutional review board of Cancer Hospital at the Chinese Academy of Medical Sciences or by the Shanghai Outdo Biotech Ethics Committee. Informed consent was obtained from the patients.

Note that full information on the approval of the study protocol must also be provided in the manuscript.

Field-specific reporting

Please select the one below that is the best fit for your research. If you are not sure, read the appropriate sections before making your selection.

☒ Life sciences ☐ Behavioural & social sciences ☐ Ecological, evolutionary & environmental sciences

For a reference copy of the document with all sections, see nature.com/documents/nr-reporting-summary-flat.pdf

Life sciences study design

All studies must disclose on these points even when the disclosure is negative.

Sample size	No statistical methods were used to determine sample size. Sample size was determined empirically or from sizes commonly published for the specific type of experiment (PMID:27642729). We normally utilized 6 mice/group or more. We used less number of mice in case of revision because of the limited duration of time. For all mouse studies, the n value corresponds to individual mice of a given treatment. For in vitro studies, all the experiments were replicated at least for 3 individual, independent experiments.
Data exclusions	No data was excluded from the manuscript
Replication	The experiments were replicated at least three times if not stated otherwise.
Randomization	For in vivo experiments, animals were randomized based on tumor burden before they were assigned into different treatment groups, to make sure the starting tumor burden in different treatment groups was similar before treatment. All groups were age and sex matched. For all other experiments, samples were allocated into experimental groups randomly.
Blinding	Investigators were not blinded as endpoint criteria of mouse experiments were defined prior to experiment. Mice reaching endpoint criteria were analyzed independently of their group allocation.

Reporting for specific materials, systems and methods

We require information from authors about some types of materials, experimental systems and methods used in many studies. Here, indicate whether each material, system or method listed is relevant to your study. If you are not sure if a list item applies to your research, read the appropriate section before selecting a response.

Materials & experimental systems

n/a	Involved in the study
☐	☒ Antibodies
☐	☒ Eukaryotic cell lines
☒	☐ Palaeontology and archaeology
☐	☒ Animals and other organisms
☒	☐ Clinical data
☒	☐ Dual use research of concern
☒	☐ Plants

Methods

n/a	Involved in the study
☒	☐ ChIP-seq
☐	☒ Flow cytometry
☒	☐ MRI-based neuroimaging

Antibodies

Antibodies used

c-MYC-monoclonal antibody (Proteintech, cat. #67447-1-Ig, clone 3D9C12), Anti-TCF7L2 antibody (Abcam, cat. #ab32873, clone 0.T.149), Anti-Id2 antibody (Santa Cruz, cat. #sc-398104, clone E-7), Snail (C15D3) rabbit mAb antibody (Cell Signaling Technology, cat. #3879S, clone N/A), ID1 polyclonal antibody (Proteintech, cat. #18475-1-AP), Anti-CD68 antibody (Abcam, cat. #ab955, clone KP1), Anti-F4/80 antibody (Abcam, cat. #ab6640), Mouse-anti-GAPDH mAb (ZSGB-BIO, cat. #TA-08, clone OTI2D9), FAK Antibody (Cell Signaling Technology, cat. #3285S), Phospho-FAK (Tyr397) antibody (Cell Signaling Technology, cat. #3283S), STAT1 polyclonal antibody (Proteintech, cat. #10144-2-AP), Phospho-Stat1 (Tyr701) (58D6) rabbit mAb (Cell Signaling Technology, cat. #9167S, clone 58D6), Lamin A/C (4C11) mouse mAb (Cell Signaling Technology, cat. #4777S, clone 4C11), Anti-GFP (rabbit) (MBL Biotech, cat. #598), HA tag polyclonal antibody (Proteintech, cat. #51064-2-AP), Anti–Myc-tag pAb (MBL Biotech, cat. #562), Anti-DDDDK-tag mAb (MBL Biotech, cat. #M185-7), DYKDDDDK tag monoclonal antibody (Proteintech, cat. # 66008-3-Ig, clone 2B3C4), Anti-CD8α (mouse specific) (Cell Signaling Technology, cat. #98941S, clone D4W2Z), STAT1 monoclonal antibody (Invitrogen, cat. #AHO0832, clone STAT1-79), Anti-STAT1 antibody [EPR23049-111] – ChIP grade (Abcam, cat. #ab239360), Anti-Ki67 antibody (Abcam, cat. #ab15580), APC anti-mouse/human CD44 antibody (Biolegend, cat. #103011, clone IM7), PE anti-mouse CD45 recombinant antibody (Biolegend, cat. #157604, clone QA17A26), APC anti-mouse CD45 antibody (Biolegend, cat. #103111, clone 30-F11), FITC anti-mouse CD45 antibody (Biolegend, cat. #157607, clone QA17A26), PE anti-mouse CD326 (Ep-CAM) Antibody (Biolegend, cat. #118205, clone G8.8), PerCP/Cyanine5.5 anti-mouse CD3ε antibody (Biolegend, cat. #100328, clone 145-2C11), APC/Cyanine7 anti-mouse CD8α antibody (Biolegend, cat. #100714, clone 53-6.7), PE anti-mouse IFN-γ antibody (Biolegend, cat. #505808, clone XMG1.2), PE/Cy7 anti-human/mouse Granzyme B recombinant antibody (Biolegend, cat. #372213, clone QA16A02), PE anti-mouse F4/80 recombinant antibody (Biolegend, cat. #157304, clone QA17A29), Anti-mouse CD3 SAFIRE purified (BioGems, cat. #05112-25, clone 17A2), Anti-Mouse CD28 SAFIRE purified (BioGems, cat. #10312-25, clone 37.51), InVivoMAb rat IgG1 isotype control (Bio X Cell, cat. #BE0088, clone HRPN), InVivoMAb anti-mouse CD8β (Bio X Cell, cat. #BE0223, clone 53-5.8), InVivoMAb anti-mouse CTLA-4 (Bio X Cell, cat. #BE0164, clone 9D9), YAP (D8H1X) XP Rabbit mAb (Cell Signaling Technology, cat. #14074, clone N/A), Anti-YAP (phospho Y357) antibody (Abcam, ab254343, clone N/A), FITC anti-mouse Lgr5 antibody (Miltenyi Biotec, cat. #130-111-393, clone DA04-10E8.9), PE anti-human LGR5 antibody (BioLegend, cat. #373803, clone SA222C5), PE-Cyanine7 anti-human CD8α antibody (BioLegend, cat. #301012, clone RPA-T8), PE anti-mouse Ly-6G/Ly-6C (Gr-1) antibody (BioLegend, cat. #108407, clone RB6-8C5). For immunoblotting, antibodies were diluted as 1:1000. For immunostaining and immunohistochemistry, antibodies were diluted as 1:100. For flow cytometry, antibodies were added 1 μL per 10^6 cell.

Validation

c-MYC-monoclonal antibody (Proteintech, cat. #67447-1-Ig, clone 3D9C12), https://www.ptglab.com/Products/MYC-Antibody-67447-1-Ig.htm, Zhu Y et al, 2022.
Anti-TCF7L2 antibody (Abcam, cat. #ab32873, Clone 0.T.149), https://www.abcam.cn/products/primary-antibodies/tcf-4tcf7l2-antibody-6h5-3-ab32873.html.
Anti-Id2 antibody (Santa Cruz, cat. #sc-398104, clone E-7), https://www.scbt.com/p/id2-antibody-e-7?requestFrom=search, Hou, H et al, 2023.
Snail (C15D3) rabbit mAb antibody (Cell Signaling Technology, cat. #3879S, clone N/A), https://www.cellsignal.com/products/primary-antibodies/snail-c15d3-rabbit-mab/3879, Mengya Guo et al, 2023.
ID1 polyclonal antibody (Proteintech, cat. #18475-1-AP), https://www.ptglab.com/products/ID1-Antibody-18475-1-AP.htm, Dong X et al, 2021.
Anti-CD68 antibody (Abcam, cat. #ab955, clone KP1), https://www.abcam.cn/products/primary-antibodies/cd68-antibody-kp1-ab955.html, Shen H et al, 2023.
Anti-F4/80 antibody (Abcam, cat. #ab6640), https://www.abcam.cn/products/primary-antibodies/f480-antibody-cia3-1-macrophage-marker-ab6640.html, Ohue-Kitano R et al, 2023.
Mouse-anti-GAPDH mAb (ZSGB-BIO, cat. #TA-08, clone OTI2D9), http://www.zsbio.com/product/TA-08, Yu JJ et al, 2020.
FAK Antibody (Cell Signaling Technology, cat. #3285S), https://www.cellsignal.cn/products/primary-antibodies/fak-antibody/3285, Mattia Saggioro et al, 2023.
Phospho-FAK (Tyr397) antibody (Cell Signaling Technology, cat. #3283S), https://www.cellsignal.cn/products/primary-antibodies/phospho-fak-tyr397-antibody/3283, Jiaxin Liang et al, 2022.
STAT1 polyclonal antibody (Proteintech, cat. #10144-2-AP), https://www.ptglab.com/products/STAT1-Antibody-10144-2-AP.htm, Shang S et al, 2022.
Phospho-Stat1 (Tyr701) (58D6) rabbit mAb (Cell Signaling Technology, cat. #9167S, clone 58D6), https://www.cellsignal.cn/products/primary-antibodies/phospho-stat1-tyr701-58d6-rabbit-mab/9167, Marco M Rodari et al, 2023.
Lamin A/C (4C11) mouse mAb (Cell Signaling Technology, cat. #4777S, clone 4C11), https://www.cellsignal.cn/products/primary-antibodies/lamin-a-c-4c11-mouse-mab/4777, Paulina Gregorczyk et al, 2023.
Anti-GFP (rabbit) (MBL Biotech, cat. #598, clone N/A), https://www.mblintl.com/products/598/, Trinh DA et al, 2019.
HA tag polyclonal antibody (Proteintech, cat. #51064-2-AP), https://www.ptglab.com/products/HA-tag-Antibody-51064-2-AP.htm, Liu Q et al, 2021.
Anti–Myc-tag pAb (MBL Biotech, cat. #562, clone N/A), https://www.mblintl.com/products/562/, Yamaguchi T et al, 2007.

Anti-DDDDK-tag mAb (MBL Biotech, cat. #M185-7, clone N/A), https://www.mblintl.com/products/m185-7/, Ding X et al, 2019.
DYKDDDDK tag monoclonal antibody (Proteintech, cat. # 66008-3-Ig, clone 2B3C4), https://www.ptglab.com/products/Flag-tag-Antibody-66008-3-Ig.htm, Mao C et al, 2021.
Anti-CD8α (mouse specific) (Cell Signaling Technology, cat. #98941S, clone D4W2Z), https://www.cellsignal.cn/products/primary-antibodies/cd8a-d4w2z-xp-rabbit-mab/98941, Atsuko Deguchi et al, 2023.
STAT1 monoclonal antibody (Invitrogen, cat. #AHO0832, clone STAT1-79), https://www.thermofisher.cn/cn/zh/antibody/product/STAT1-Antibody-clone-STAT1-79-Monoclonal/AHO0832, Kim DW et al, 2022.
Anti-STAT1 antibody [EPR23049-111] – ChIP grade (Abcam, cat. #ab239360), https://www.abcam.com/products/primary-antibodies/stat1-antibody-epr23049-111-chip-grade-ab239360.html, Wang J et al, 2022.
Anti-Ki67 antibody (Abcam, cat. #ab15580, polyclonal), https://www.abcam.com/products/primary-antibodies/ki67-antibody-ab15580.html, Sun XC et al, 2023.
APC anti-mouse/human CD44 antibody (Biolegend, cat. #103011, clone IM7), https://www.biolegend.com/en-us/products/apc-anti-mouse-human-cd44-antibody-312, Cignarella F et al, 2018.
PE anti-mouse CD45 recombinant antibody (Biolegend, cat. #157604, clone QA17A26), https://www.biolegend.com/en-us/products/pe-anti-mouse-cd45-recombinant-antibody-18946, Podd BS et al, 2006.
APC anti-mouse CD45 antibody (Biolegend, cat. #103111, clone 30-F11), https://www.biolegend.com/en-us/products/apc-anti-mouse-cd45-antibody-97, Cha JH et al, 2018.
FITC anti-mouse CD45 antibody (Biolegend, cat. #157607, clone QA17A26), https://www.biolegend.com/en-us/products/fitc-anti-mouse-cd45-recombinant-antibody-18940, Zeng Z et al, 2022.
PE anti-mouse CD326 (Ep-CAM) Antibody (Biolegend, cat. #118205, clone G8.8), https://www.biolegend.com/en-us/products/pe-anti-mouse-cd326-ep-cam-antibody-4726, Kimura S et al, 2020.
PerCP/Cyanine5.5 anti-mouse CD3ε antibody (Biolegend, cat. #100328, clone 145-2C11), https://www.biolegend.com/en-us/products/percp-cyanine5-5-anti-mouse-cd3epsilon-antibody-4191, Duarte J et al, 2012.
APC/Cyanine7 anti-mouse CD8α antibody (Biolegend, cat. #100714, clone 53-6.7), https://www.biolegend.com/en-us/products/apc-cyanine7-anti-mouse-cd8α-antibody-2269, Cha JH et al, 2018.
PE anti-mouse IFN-γ antibody (Biolegend, cat. #505808, clone XMG1.2), https://www.biolegend.com/en-us/products/pe-anti-mouse-ifn-gamma-antibody-997, Wu MJ et al, 2022.
PE/Cy7 anti-human/mouse Granzyme B recombinant antibody (Biolegend, cat. #372213, clone QA16A02), https://www.biolegend.com/en-us/products/pe-cyanine7-anti-humanmouse-granzyme-b-recombinant-antibody-15582, Cho JW et al, 2021.
PE anti-mouse F4/80 recombinant antibody (Biolegend, cat. #157304, clone QA17A29), https://www.biolegend.com/en-us/products/pe-anti-mouse-f4-80-recombinant-antibody-18755, Cai B et al, 2021.
Anti-mouse CD3 SAFIRE purified (BioGems, cat. #05112-25, clone 17A2), https://www.bio-gems.com/anti-mouse-cd3-safire-purified.html, Miescher, G et al, 1989.
Anti-Mouse CD28 SAFIRE purified (BioGems, cat. #10312-25, clone 37.51), https://www.bio-gems.com/anti-mouse-cd28-safire-purified.html, Nandi, D et al, 1994.
InVivoMAb rat IgG1 isotype control (Bio X Cell, cat. #BE0088, clone HRPN), https://bioxcell.com/invivomab-rat-igg1-isotype-control-anti-horseradish-peroxidase-be0088, Goschl, L., et al, 2018.
InVivoMAb anti-mouse CD8β (Bio X Cell, cat. #BE0223, clone 53-5.8), https://bioxcell.com/invivomab-anti-mouse-cd8b-lyt-3-2-be0223, Howland, S. W., et al, 2015.
InVivoMAb anti-mouse CTLA-4 (Bio X Cell, cat. #BE0164, clone 9D9), https://bioxcell.com/invivomab-anti-mouse-ctla-4-cd152-be0164, Dai, M., et al, 2015.
YAP (D8H1X) XP Rabbit mAb (Cell Signaling Technology, cat. #14074, clone N/A), https://www.cellsignal.cn/products/primary-antibodies/yap-d8h1x-xp-rabbit-mab/14074, Li L et al, 2023.
Anti-YAP (phospho Y357) antibody (Abcam, ab254343, clone N/A), https://www.abcam.cn/products/primary-antibodies/yap1-phospho-y357-antibody-epr23680-48-ab254343.html?productWallTab=ShowAll, Silva J et al, 2022.
FITC anti-mouse Lgr5 antibody (Miltenyi Biotec, cat. #130-111-393, Clone DA04-10E8.9), https://www.miltenyibiotec.com/CN-en/products/lgr5-antibody-anti-mouse-da04-10e8-9.html#conjugate=vio-bright-fitc:size=30-ug-in-200-ul, Morita, H. et al, 2004.
PE anti-human LGR5 antibody (BioLegend, cat. #373803, clone SA222C5), https://www.biolegend.com/en-us/products/pe-anti-human-lgr5-gpr49-antibody-14385.
PE-Cyanine7 anti-human CD8α antibody (BioLegend, cat. #301012, clone RPA-T8), https://www.biolegend.com/en-us/products/pe-cyanine7-anti-human-cd8a-antibody-838, Carpenter RS et al, 2019.
PE anti-mouse Ly-6G/Ly-6C (Gr-1) antibody (BioLegend, cat. #108407, clone RB6-8C5), https://www.biolegend.com/en-us/products/pe-anti-mouse-ly-6g-ly-6c-gr-1-antibody-460, Chinta KC et al, 2018.

Eukaryotic cell lines

Policy information about cell lines and Sex and Gender in Research

Cell line source(s)	Human CRC cell lines HCT-116, DLD-1 and HCT-8; mouse tumor cell lines H22, Pan02, MC38 and CT26; mouse monocytic/macrophage cell line RAW 264.7; human monocytic cell line THP-1; and human embryonic kidney 293T (HEK 293T) cells were purchased from the cell culture center of Peking Union Medical College.
Authentication	Human cell lines were authenticated by STR.
Mycoplasma contamination	All cell lines in our laboratory are routinely tested for mycoplasma contamination and cells used in this study are negative for mycoplasma.
Commonly misidentified lines (See ICLAC register)	No cell line used in the paper is listed in ICLAC database.

Animals and other research organisms

Policy information about studies involving animals; ARRIVE guidelines recommended for reporting animal research, and Sex and Gender in Research

Laboratory animals	C57BL/6J mice (female, male, 6-8 weeks old), BALB/c mice (male, 6-8 weeks old), BALB/c-Foxn1nu/Nju (named as BALB/c-nude hereafter) mice (male, 6 weeks old) were purchased from the BEIJING HFK BIOSCIENCE CO., LTD. OT1 and Lyz2tm1(cre)Ifo/J mice were provided by Cyagen Biosciences Inc. Id1f/f mice were generated by Cyagen Biosciences Inc. Myeloid cell-lineage-specific Id1 deficient mice (Id1Lyz-ko) were generated by crossing Id1f/f mice with Lyz2tm1(cre)Ifo/J mice. Animals were maintained under a specific pathogen-free (SPF) facility with an appropriately controlled environment (12 h light /12 h dark cycle with temperature of18-23°C) and humidity (50-70%) an id with easy access to food and water.
Wild animals	The study did not involve wild animals.
Reporting on sex	Our data suggested that the tumor promoting role of ID1 in TAMs has no difference between sex.
Field-collected samples	No aminals were from field.
Ethics oversight	All animal procedures were conducted in accordance with the guidelines of the Institutional Committee in the Institute of Materia Medica, Chinese Academy of Medical Sciences & Peking Union Medical College and Chinese Center for Disease Control and Prevention for the Ethics of Animal Care and Treatment. The animal study also accorded with the ARRIVE guidelines. According to the requirments of ethics committee, the maximal tumor sizes are all less than 2000 mm3. In some cases, this limit has been exceeded the last day of measurement and the mice were immediately euthanized.

Note that full information on the approval of the study protocol must also be provided in the manuscript.

Plants

Seed stocks	N/A
Novel plant genotypes	N/A
Authentication	N/A

Flow Cytometry

Plots

Confirm that:

- [x] The axis labels state the marker and fluorochrome used (e.g. CD4-FITC).
- [ ] The axis scales are clearly visible. Include numbers along axes only for bottom left plot of group (a 'group' is an analysis of identical markers).
- [x] All plots are contour plots with outliers or pseudocolor plots.
- [x] A numerical value for number of cells or percentage (with statistics) is provided.

Methodology

Sample preparation	Flow cytometry for adherent cells:<br>For surface staining, cells were stained with antibodies conjugated with fluorochromes after FcR block.<br><br>Flow cytometry for tumor tissues:<br>Single-cell suspensions were prepared from fresh tissues and enriched by density gradient centrifugation.<br>For surface staining, cells were stained with antibodies conjugated with fluorochromes after FcR block.<br>For intracellular cytokine staining, cells were incubated in culture medium containing PMA (5 ng/ml), Ionomycin (500 ng/ml), Brefeldin A (1: 1000) and Golgistop (1: 1000) at 37°C for 4 hours. Subsequent surface staining and intracellular staining were performed using Cytofix/Cytoperm™ fixation/permeabilization solution kit (BD Biosciences, 554714).
Instrument	Data collection and analysis was performed on BD FACS Verse.
Software	FCS Express 6 software

Cell population abundance	When cells were sorted or enriched, the purity was confirmed by flow cytometry and in each case the purity was above 90% .
Gating strategy	The gating strategy was supported in the Supplementary Fig. 9.

☒ Tick this box to confirm that a figure exemplifying the gating strategy is provided in the Supplementary Information.
