# Supplementary figures and images for: Author Correction: ID1 expressing macrophages support cancer cell stemness and limit CD8+ T cell infiltration in colorectal cancer
Source: Nat Commun. 2026 Feb 9;17:1444. doi: 10.1038/s41467-026-69408-x (PMC12887014; doi:10.1038/s41467-026-69408-x)

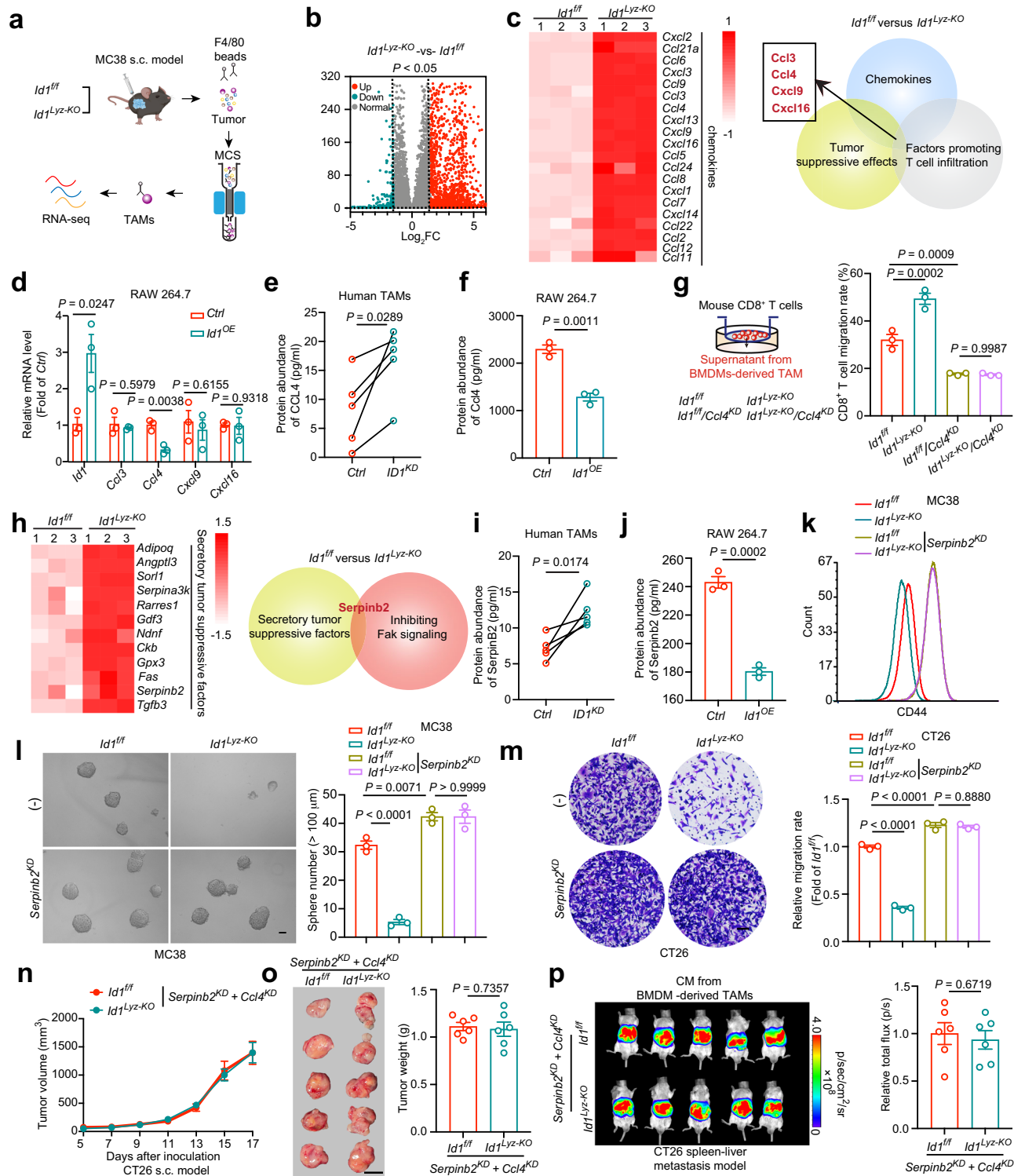

Supplement: Supplementary file 1 — Original, uncorrected Fig. 5 [file 41467_2026_69408_MOESM1_ESM.pdf]
